# Evaluation of Biocontrol Efficacy of *Bacillus velezensis* HAB-2 Combined with *Pseudomonas hunanensis* and *Enterobacter soli* Against Cowpea Fusarium Wilt

**DOI:** 10.3390/microorganisms13112578

**Published:** 2025-11-12

**Authors:** Wei Wei, Tianlong Qi, Jinpeng Lu, Xi Wei, Peilin Wu, Justice Norvienyeku, Weiguo Miao, Wenbo Liu

**Affiliations:** 1 Key Laboratory of Green Prevention and Control of Tropical Plant Diseases and Pests (Ministry of Education), School of Tropical Agriculture and Forestry, Hainan University, Haikou 570228, China; 24220951320192@hainanu.edu.cn (W.W.); 22220951320049@hainanu.edu.cn (T.Q.); 23220951320084@hainanu.edu.cn (J.L.); 22210904000032@hainanu.edu.cn (X.W.); 24220951320196@hainanu.edu.cn (P.W.); jk_norvienyeku@hainanu.edu.cn (J.N.);; 2 Danzhou Invasive Species Observation and Research Station of Hainan Province, Hainan University, Danzhou 571799, China

**Keywords:** disease suppression, biocontrol, cowpea *Fusarium* wilt, plant growth-promoting rhizobacteria

## Abstract

Cowpea Fusarium wilt (CFW) is a soilborne fungal disease caused by *Fusarium oxysporum* f. sp. *tracheiphilum* (Fot), leading to substantial yield losses globally. This study evaluates the biocontrol potential of *Bacillus velezensis* HAB-2 and develops a microbial combination for effective disease management. *B. velezensis* HAB-2 suppressed *F. oxysporum* f. sp. *tracheiphilum* AIQBFO93 growth by 69.8% in vitro and exhibited multiple plant growth-promoting traits. Pot experiments demonstrated that HAB-2 alone achieved a 47.62% control rate against CFW. Furthermore, two compatible plant growth-promoting rhizobacteria (PGPR), *Pseudomonas hunanensis* HD33 and *Enterobacter soli* HD42, were isolated from the rhizosphere soil of cowpea previously treated with HAB-2. These two strains were combined with HAB-2 at different concentrations in 15 microbial combinations. The combined application of the three strains provided more consistent disease control, with the optimal combination demonstrating a 15.15% higher control rate than HAB-2 alone. Compared to the untreated control, this combination significantly increased cowpea fresh weight, leaf area, and plant height by 10.60%, 8.04%, and 7.81%, respectively, and upregulated the expression of defense-related genes, indicating enhanced resistance. These results confirm that *B. velezensis* HAB-2 is an effective biocontrol agent against wilt disease, and its synergistic application with functionally complementary PGPR strains provides a viable strategy for sustainable crop disease management.

## 1. Introduction

Cowpea [*Vigna unguiculata* (L.) Walp.] is an annual herbaceous plant in the *Vigna* genus of the Fabaceae family. Widely cultivated in tropical and subtropical regions, it holds substantial nutritional and economic value [1]. As a key leguminous crop, cowpea is grown on approximately 14.5 million hectares worldwide, with an annual production of around 6.5 million tons. It is used as an essential source of dietary protein in many developing countries [2,3]. However, continuous monoculture of cowpea often leads to severe disease outbreaks, with *Fusarium* wilt being particularly devastating [4]. This disease, known as cowpea *Fusarium* wilt(CFW), is caused by *Fusarium oxysporum* f. sp. *tracheiphilum* (Fot) and is a prevalent soil-borne disease affecting global cowpea production [5,6]. As a hemibiotrophic pathogen, *F. oxysporum* infects host plants through wounds in the root system, causing basal stem swelling, leaf yellowing and wilting, vascular discoloration, and ultimately death. Yield losses can reach as 70% [4]. Fot produces chlamydospores that enter a dormant state under unfavorable conditions, enhancing its environmental adaptability and survival in soil for several years [7]. Consequently, effective control of CFW remains a significant challenge in cowpea production.

The primary strategies for managing CFW include cultivating resistant varieties and applying chemical fungicides. Traditional breeding methods necessitate comprehensive screening of germplasm resources, followed by the integration of resistance traits into target cultivars, a process that typically spans several years [8]. Additionally, pathogenic fungi can evolve to overcome host resistance, leading to a loss of resistance in previously effective germplasm [9]. Prolonged reliance on chemical fungicides not only poses a risk of exceeding residue limits [10,11,12] but also presents environmental threats and harm to non-target organisms [13]. Certain fungicide compounds have been linked to carcinogenicity, teratogenicity, and mutagenicity in humans [14]. Therefore, to ensure the sustainable development of the cowpea industry, it is crucial to explore sustainable disease management strategies that balance ecological safety with effective disease control. Biological control is widely considered a safe, environmentally friendly, and sustainable approach to managing plant diseases [15,16]. *Bacillus velezensis*, exhibiting strong environmental adaptability and a broad spectrum of ecological niches, is a promising candidate for biological control. It can be sourced from various locations such as soil, the rhizosphere, and internal plant tissues [17,18,19]. In an agricultural context, *B. velezensis* has been effectively employed as both a biofungicide and biofertilizer [20,21]. A multitude of studies corroborate its effectiveness in inhibiting *Fusarium* wilt in various economically important crops, including cucumber, banana, watermelon, tomato, and others [22,23,24]. The antagonistic activity of *B. velezensis* against plant pathogens involves multiple mechanisms. It synthesizes a wide array of antimicrobial compounds—such as volatile organic compounds (VOCs), bacteriocins, hydrolytic enzymes, polyketides (PKs), and cyclic lipopeptides (CLiPs)—that serve to exert direct antagonistic effects on pathogen growth. Moreover, *B. velezensis* enhances plant resistance by promoting growth and triggering the expression of defense-related genes [25].

*Bacillus* species, key members of the plant growth-promoting rhizobacteria (PGPR), exhibit the common characteristic of attracting beneficial microbes to the root zone, thereby enhancing plant growth and suppressing pathogens [26]. The co-application of PGPR strains not only strengthens antagonistic activity against phytopathogens but also induces plant resistance, leading to improved disease control efficacy [27,28]. Compared to inoculation with a single strain, strategically designed PGPR consortia can activate a broader range of induced systemic resistance pathways, achieving over 50% disease suppression against multiple pathogens. This significantly broadens the biocontrol spectrum beyond that of individual strains [29]. Compared to single-strain inoculation, an assemblage of eight *Pseudomonas fluorescens* strains can sustain a high colonization density in the rhizosphere, thereby ensuring efficient pathogen suppression during the later stages of infection. Under greenhouse conditions, this consortium assembly exhibits augmented control efficacy against tomato bacterial wilt induced by *Ralstonia solanacearum* [30]. Compound microbial agents have also proven superior to single strains in managing banana *Fusarium* wilt. The four-strain combination T28 consistently suppresses disease across diverse soil conditions, achieving a field control efficacy of 57.14% [31]. Research on biocontrol bacterial resources for CFW remains limited. This study first verified the antifungal activity of *B. velezensis* strain HAB-2 against *F. oxysporum* f. sp. *tracheiphilum* AIQBFO93 and its plant growth-promoting properties, and evaluated its effects on CFW and plant growth through pot experiments. The activities of catalase (CAT), peroxidase (POD), and superoxide dismutase (SOD) in cowpea leaves were measured to analyze the regulatory effects of HAB-2 on plant defense enzymes. In addition, two PGPR strains—*P. hunanensis* HD33 and *Enterobacter soli* HD42 were isolated from the potting soil. Their combined application with HAB-2 was further investigated to assess their effects on disease suppression and the expression of defense-related genes in cowpea. Although previous studies have confirmed the biocontrol potential of *B. velezensis* against *Fusarium* pathogens, its combined application with functionally complementary PGPR in cowpea remains relatively underexplored. By integrating newly isolated rhizobacterial strains with HAB-2, this study developed a microbial consortium strategy that not only enhanced disease suppression but also promoted plant growth and activated host defense responses, thereby broadening the research perspective for sustainable management of CFW.

## 2. Materials and Methods

### 2.1. Strains, Plant Materials, and Culture Conditions

The strains *B. velezensis* HAB-2, HAB-2-*gfp*, and *F. oxysporum* f. sp. *tracheiphilum* AIQBFO93 (GenBank: PV178668.1) were provided by the Key Laboratory of Green Prevention and Control of Tropical Plant Diseases and Pests (Ministry of Education), Hainan University, Danzhou, Hainan Province, China. The cowpea (*Vigna unguiculata*) cultivar ‘Texuan Zhangtangwang’ was purchased from Beijing Sibeiqi Seed Co., Ltd. (Beijing, China) Sterile soil preparation: Potting soil was collected from the Plant Protection Base at Hainan University, Danzhou Campus, and thoroughly mixed with organic fertilizer, vermiculite, and coconut coir at a volume ratio of 2:1:1:1. The mixture was sterilized at 121 °C for 20 min and stored for later use.

The single colony of HAB-2 was inoculated into Luria–Bertani (LB, 10 g of tryptone, 5 g of yeast extract, 10 g of NaCl, per liter, 1000 mL of distilled water, pH = 7.0–7.2) broth and cultured at 28 °C with continuous agitation at 180 rpm for 48 h. The bacterial concentration at OD_600_ = 1 was determined to be 1 × 10^9^ CFU/mL using a pre-established OD_600_–CFU calibration curve (spread plate method). Serial dilutions were performed to obtain suspensions of desired concentrations. *F. oxysporum* f.sp. *tracheiphilum* AIQBFO93 was cultured in potato dextrose broth (PDB) at 28 °C with shaking at 180 rpm for 3 days. Mycelia were filtered through sterile gauze, and the spore suspension was adjusted to 1 × 10^6^ CFU/mL using a hemocytometer.

### 2.2. In Vitro Screening of Plant Growth-Promoting Traits

#### 2.2.1. Nitrogen Fixation and Solubilization of Phosphorus and Potassium

The isolates were inoculated onto specific screening media to evaluate their nitrogen fixation, potassium solubilization, and phosphate solubilization abilities [32,33]. Nitrogen fixation ability was assessed using nitrogen-free agar medium (NFM). Growth on NFM was observed exclusively in strains possessing the capability of nitrogen fixation. Bacterial strains were separately inoculated onto potassium feldspar agar medium (PF), MeHKNHa inorganic medium (PVK), and organic phosphorus medium (MOP). The isolates capable of solubilizing phosphate or potassium were identified by the presence of translucent halos surrounding the colonies on the media after incubation.

#### 2.2.2. Siderophore Production Assay

Siderophore production by bacterial isolates was evaluated using the modified Chrome Azurol S (CAS) agar medium Kit (PM0821, Beijing Coolaber Technology Co., Ltd., Beijing, China). According to the manufacturer’s instructions, a dual-layer agar medium method was employed. The bottom layer consisted of 10 mL of water agar mixed with CAS detection solution, while the top layer contained 10 mL of basal medium supplemented with buffer solution. 5 μL of bacterial suspension was placed on CAS agar medium. After incubation at 28 °C for 5 days, the appearance of yellow-orange halos surrounding the colonies was considered indicative of siderophore production [34].

#### 2.2.3. Indole-3-Acetic Acid Production Assay

To establish the standard curve for IAA quantification, 250 μL of IAA standard solutions (0, 5, 10, 20, 40, and 60 μg/mL) were each mixed with 1 mL of Salkowski colorimetric solution (PH1941, PHYGENE, Fuzhou, China). The mixtures were incubated in the dark for 30 min, and absorbance was measured at 530 nm using a spectrophotometer. For preliminary screening of IAA-producing strains, bacterial isolates were inoculated into LB broth supplemented with 100 μg/mL L-tryptophan and incubated at 28 °C with shaking at 180 rpm for 48 h. Subsequently, 1 mL of the culture was centrifuged at 8000 rpm for 5 min. A volume of 50 μL of the supernatant was mixed with 50 μL of Salkowski solution in a 96-well microplate and incubated at room temperature in the dark for 30 min. The development of a pink coloration indicated the presence of IAA. Strains that tested positive for IAA production were further quantified by measuring absorbance at 530 nm under the same conditions used for the standard curve. The IAA concentration was calculated based on the corresponding absorbance values using the established standard curve.

### 2.3. Assessment of Extracellular Enzyme Activities of B. velezensis HAB-2

The ability of *B. velezensis* HAB-2 to secrete extracellular enzymes was assessed by culturing the strain on test agar plates. Protease activity was examined using agar medium supplemented with 1% (*w*/*v*) skim milk [35]. Chitinase production was determined on colloidal chitin medium [36]. The strain was inoculated onto the screening medium and incubated at 28 °C for 2 days. Clear zones around colonies indicated protease and chitinase production by the strain.

To assess cellulase production, HAB-2 was cultured on solid medium containing 0.1% (*w*/*v*) sodium carboxymethyl cellulose (CMC-Na) for 2 days at 28 °C [37]. The plates were then flooded with 5 mL of 0.1% (*w*/*v*) Congo red solution for 20 min, and subsequently decolorized with 10 mL of 1 mol/L sodium chloride solution for 5 min. The presence of hydrolysis zones surrounding the colonies was used to evaluate the cellulase-producing capacity of the strain.

### 2.4. Inhibitory Effect of B. velezensis HAB-2 on F. oxysporum f. sp. tracheiphilum AIQBFO93

The antagonistic effect of strain HAB-2 against *F. oxysporum* f. sp. *tracheiphilum* AIQBFO93 was evaluated using a dual-culture assay [38]. A 5.0 mm mycelial plug of the pathogen was placed at the center of a potato dextrose agar (PDA) plate. Bacterial suspension (5 μL of 10^8^ CFU/mL HAB-2) was spot-inoculated at four points 2.5 cm from the center along perpendicular axes and air-dried. Plates inoculated with 5 μL of LB liquid medium served as negative controls. After incubation at 28 °C for 7 days, the diameter of the fungal colony was measured. The inhibition rate of HAB-2 against the pathogen was calculated using the following formula [39]:

Inhibition rate (%) = [(Colony diameter of the control − Colony diameter of the treatment)/Colony diameter of the control] × 100%.(1)

The impact of *B. velezensis* HAB-2 on the hyphal morphology and sporulation of *F. oxysporum* f. sp. *tracheiphilum* AIQBFO93 was evaluated using a liquid co-culture method described by Soni [40], with several modifications. A mycelial plug of the pathogen was inoculated into PDB and incubated at 28 °C with agitation at 180 rpm for 5 days. The culture was diluted with sterile water to a spore concentration of 1 × 10^9^ spores/mL. Subsequently, 1 mL of this suspension was transferred into 150 mL of PDB, and 1 mL of HAB-2 bacterial suspension (1 × 10^9^ CFU/mL) was added. The control group consisted of 1 mL of pathogen suspension mixed with 1 mL of LB liquid medium. After incubation at 28 °C and 180 rpm for 24 h, hyphal and spore morphology were observed under a light microscope.

### 2.5. Colonization of B. velezensis HAB-2-GFP on Cowpea Roots

Cowpea seedlings were gently rinsed with sterile water to remove soil particles from the root surface. The roots were then immersed in *B. velezensis* HAB-2-GFP (1 × 10^9^ CFU/mL) for 20 min. After treatment, the seedlings were transplanted into Hoagland’s nutrient solution. At 24 h post-inoculation, root samples were collected and sectioned both transversely and longitudinally using a sterile blade. Colonization of HAB-2-GFP was observed under a fluorescence microscope.

### 2.6. Evaluation of the Biocontrol Efficacy of HAB-2 Against Cowpea Fusarium Wilt

The surface-sterilized seeds were soaked in sterile water at 50 °C for 50 min and subsequently transferred to Petri dishes for germination. Germinated seeds were transferred to seedling trays containing sterilized soil and cultivated in a greenhouse (Temperature was maintained at 26 ± 2 °C, with a relative humidity of 65%. Light intensity was set at 5800 LUX, and the photoperiod was set to 12 h of light followed by 12 h of darkness each day). Healthy seedlings were selected for subsequent experiments. After 10 days of growth, healthy cowpea seedlings were selected and transplanted into pots following root tip excision. Inoculation of *F. oxysporum* f. sp. *tracheiphilum* AIQBFO93 was carried out by thoroughly mixing the pathogen into the soil. Before transplantation, 100 mL of a *F. oxysporum* f. sp. *tracheiphilum* AIQBFO93 spore suspension (1 × 10^5^ CFU/mL) was thoroughly mixed into sterile soil. An equal volume of sterile water was used as the blank control. The pot experiment comprised eight treatments: (1) sterile water (W), (2) LB medium (LB), (3) pathogen + sterile water (F + W), (4) pathogen + LB medium (F + LB), (5–7) pathogen + HAB-2 at 10^9^, 10^8^, and 10^7^ CFU/mL (F + 10^9^H, F + 10^8^H, F + 10^7^H), and (8) pathogen + hymexazol (F + Y; Macklin, Shanghai, China). Each treatment group received applications at 7-day intervals, with 10 mL of the corresponding solution mixed with 40 mL of sterile water and inoculated near the root zone of cowpea seedlings. Each treatment was performed with three replicates, and each replicate consisted of three cowpea seedlings.

After the final application, plant height, root length, and fresh weight were measured. Subsequently, leaf samples from each treatment group were collected to determine the contents of ascorbic acid (VC) and soluble sugars [41,42], as well as the activities of nitrate reductase (NR), catalase (CAT), peroxidase (POD), and superoxide dismutase (SOD) [43,44]. The determinations of VC content, soluble sugar content, and nitrate reductase activity in cowpea leaves were performed with three biological replicates. Assays for defense-related enzyme (CAT, POD, SOD) activities employed five biological replicates. Meanwhile, starting from day 15 after inoculation, the disease index (DI) and control efficacy (CE) were calculated according to the grading criteria described by Rigert et al. [45].

DI = ∑(Number of plants per severity grade × Level)/((Total number of assessed plants × Maximum level) × 100%.(2)

CE = [(Disease index in control group − Disease index in treatment group)/Disease index in control group] × 100%.(3)

### 2.7. Isolation and Identification of Rhizobacteria from HAB-2-Treated Soil

Isolation of soil bacteria was performed with slight modifications to the method described by Kumar et al. [46]. Briefly, 5 g of soil was mixed with 50 mL of sterile water and shaken to release rhizosphere-associated microbes. The resulting suspension was incubated at 28 °C with shaking at 180 rpm for 1 h. Serial dilutions of the supernatant were prepared to obtain 10^−4^, 10^−5^, and 10^−6^ dilution levels. Aliquots of 100 μL from each dilution were spread onto LB agar plates and incubated at 28 °C in the dark for 3 days.

Single colonies were selected for 16S rRNA amplification using universal primers 27F and 1492R [47]. PCR reactions were performed in a 25 μL system containing 12.5 μL of enzyme mix (TIANGEN BIOTECH, Beijing, China), 9.5 μL of sterile ddH_2_O, 1 μL of bacterial suspension, and 1 μL of each primer. Thermal cycling conditions followed the manufacturer’s protocol with a modified initial denaturation step extended to 10 min.

PCR products were sequenced by BGI Genomics (Shenzhen, China) and compared with sequences in the GenBank database for homology analysis. To determine the taxonomic status of the isolates, phylogenetic analysis was conducted based on their 16S rRNA sequences using MEGA software (v.7.2). Multiple sequence alignment was performed using the MUSCLE algorithm, and a phylogenetic tree was constructed using the maximum likelihood method with the Hasegawa-Kishino-Yano (HKY) model plus invariant sites (I). The reliability of clades was tested using the 1000 bootstrap replications.

### 2.8. Evaluation of Biocontrol Efficacy of Microbial Combination Against Cowpea Fusarium Wilt

To evaluate the biocontrol efficacy of *B. velezensis* HAB-2 in combination with two rhizobacterial isolates (*P. hunanensis* HD33 and *E. soli* HD42) against *Fusarium* wilt in cowpea, a pot experiment was conducted under natural light and ambient temperature to simulate field-like conditions. The experiment was conducted under natural conditions at the Plant Protection Base of Hainan University, Danzhou Campus, from September to October 2024. Daytime temperatures ranged from 28 to 33 °C, while nighttime temperatures were between 20 and 25 °C. Relative humidity fluctuated between 70% and 90%, and the daily photoperiod averaged 11–12 h. A total of 21 treatment groups were established, including HAB-2 alone (1 × 10^9^ CFU/mL), six combinations of HAB-2 with either HD33 or HD42 at three concentration gradients (1 × 10^5^, 1 × 10^7^, and 1 × 10^9^ CFU/mL), and nine combinations involving all three strains at varying concentrations. Additional treatments included chemical fungicide (hymexazol), sterile water, and LB liquid medium. Non-inoculated controls consisted of sterile water and LB treatments. Each treatment was replicated three times, with three cowpea seedlings per replicate. Disease severity index and biocontrol efficacy were assessed 21 days post-inoculation according to the formulas described previously.

### 2.9. Analysis of Defense Gene Expression in Cowpea Leaves

To investigate the expression of defense-related genes in cowpea leaves under the most effective biocontrol treatment from the combination experiment, quantitative retro transcribed PCR was performed to quantify the expression levels of six defense-related genes in cowpea leaves: *cat*, *pod*, *sod*, *npr*, *pr1*, and *pr3*. The cowpea elongation factor 1-alpha (*EF1-α*) gene was used as the internal reference for normalization. Total RNA was extracted from cowpea leaves using the SteadyPure Plant RNA Quick Extraction Kit Kit (Accurate Biotechnology, Changsha, China),following the manufacturer’s instructions without modification. First-strand cDNA was synthesized using the ToloScript RT EasyMix for qPCR kit (Tolo Biotech, Shanghai, China), and quantitative retro transcribed PCR was conducted on an Mx3000P system (Agilent Technologies, CA, USA) using 2×Q3 SYBR qPCR Master Mix (Tolo Biotech, Shanghai, China). The thermal cycling conditions were as follows: initial denaturation at 95 °C for 30 s, followed by 40 cycles of 95 °C for 10 s, 56 °C for 30 s, and 72 °C for 30 s. A melting curve analysis was performed from 55 °C to 95 °C to verify amplification specificity, according to the instrument’s protocol. Relative gene expression levels were calculated using the 2^−ΔΔCt^ method [48]. The quantitative retro transcribed PCR primers were designed using Premier 5 (Table 1).

### 2.10. Statistical Analysis

Significant differences among treatments were assessed by one-way analysis of variance (ANOVA) followed by Duncan’s test (*p* < 0.05). Statistical analyses were performed using SPSS 26.0 software (IBM Corporation, Armonk, NY, USA). Graphs were generated using GraphPad Prism 10.1.2 software (GraphPad Software, San Diego, CA, USA).

## 3. Results

### 3.1. In Vitro Evaluation of Plant Growth-Promoting and Biocontrol Activities of B. velezensis HAB-2

**Figure 1:**
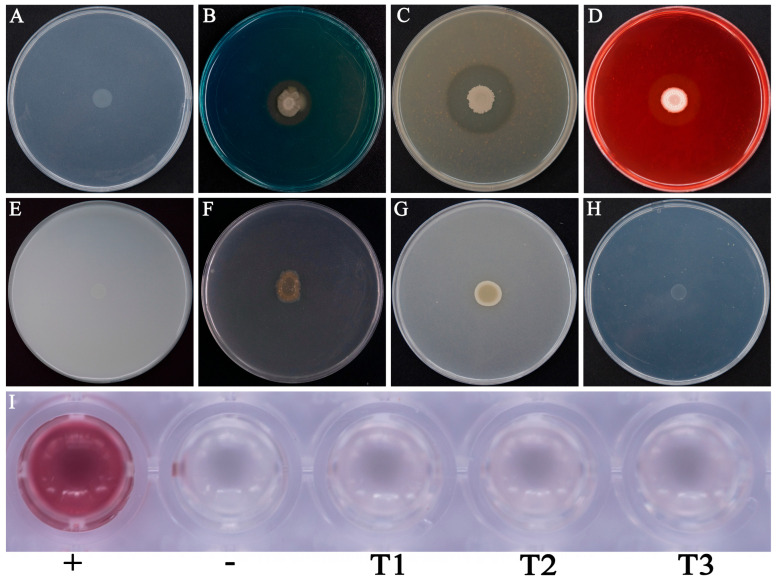
Plant growth-promoting traits and extracellular enzyme activities of *B. velezensis* HAB-2. (**A**) Nitrogen fixation; (**B**) Siderophore production; (**C**) Protease activity; (**D**) Cellulase activity; (**E**) Inorganic phosphate solubilization; (**F**) Organic phosphate solubilization; (**G**) Potassium solubilization; (**H**) Chitinase activity; (**I**) IAA production. +, indicates the positive control; −, indicates the negative control; T1–T3 represent treatment groups.

HAB-2 exhibited various growth-promoting traits, including nitrogen fixation and siderophore production. In addition, it showed extracellular enzymatic activities such as protease and cellulase synthesis (Figure 1A–D). However, no detectable activity was observed for phosphate solubilization, potassium release, chitinase, and IAA production (Figure 1E–I).

**Figure 2:**
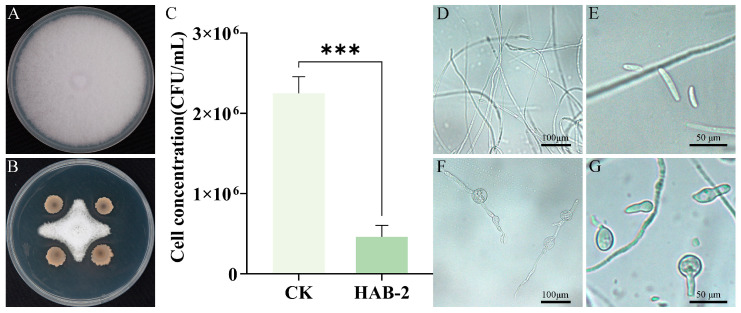
Antifungal effects of *B. velezensis* HAB-2 against Fot. (**A**) *F. oxysporum* f. sp. *tracheiphilum* AIQBFO93 growth in control; (**B**) Suppressed mycelial growth following HAB-2 treatment; (**C**) Significant reduction in spore production after HAB-2 treatment; (**D**) Normal mycelial morphology of *F. oxysporum* f. sp. *tracheiphilum* AIQBFO93; (**E**) Abnormal mycelial morphology of *F. oxysporum* f. sp. *tracheiphilum* AIQBFO93 induced by HAB-2; (**F**) Normal spores of *F. oxysporum* f. sp. *tracheiphilum* AIQBFO93; (**G**) Deformed spores following HAB-2 treatment.“***” indicates a statistically significant difference at *p* < 0.001 based on ANOVA.

The dual-culture assay revealed that *B. velezensis* HAB-2 exhibited significant antagonistic activity against Fot, with an inhibition rate of 69.8 ± 0.28% (Figure 2A,B). After 12 h of co-cultivation in PDB, the spore concentration in the HAB-2 treatment group was only 20.4% of that in the control (Figure 2C). Under microscopic observation, untreated hyphae and spores of *F. oxysporum* f. sp. *tracheiphilum* AIQBFO93 were slender and structurally intact (Figure 2D,E). Conversely, HAB-2-exposed hyphae and spores displayed pronounced morphological alterations, including hyphal distortion and swollen structures (Figure 2F,G).

**Figure 3:**
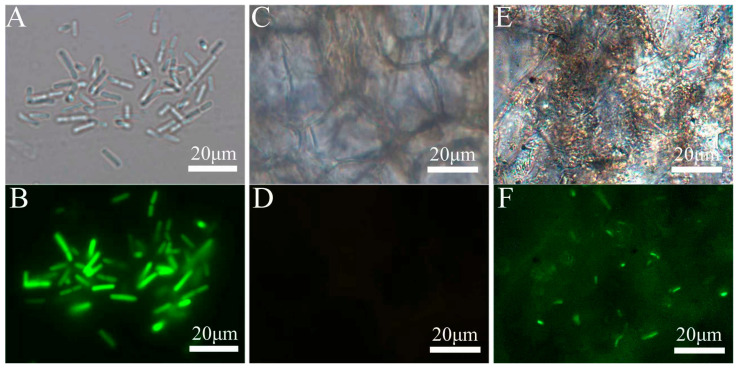
Colonization of cowpea roots by HAB-2-*gfp*. (**A**,**B**) HAB-2-*gfp* strain; (**C**,**D**) Cowpea root epidermis untreated with HAB-2-; (**E**,**F**) Cowpea root epidermis treated with HAB-2-*gfp*; (**A**,**C**,**E**) Bright-field images; (**B**,**D**,**F**) Dark-field fluorescence images.

HAB-2-*gfp* was an efficient colonizer in cowpea plants, as confirmed by observation using a fluorescence microscope for HAB-2-*gfp* in the roots of plants. The HAB-2-*gfp* strain was activated and observed under fluorescence microscopy, showing a distinct green fluorescent signal (Figure 3A,B). Compared to the control group (Figure 3C,D), green fluorescence was observed in the epidermis (Figure 3E,F) after 24 h of treatment with the HAB-2-*gfp* strain, indicating successful root colonization.

### 3.2. Biocontrol Efficacy of B. velezensis HAB-2 Against Cowpea Fusarium Wilt

**Figure 4:**
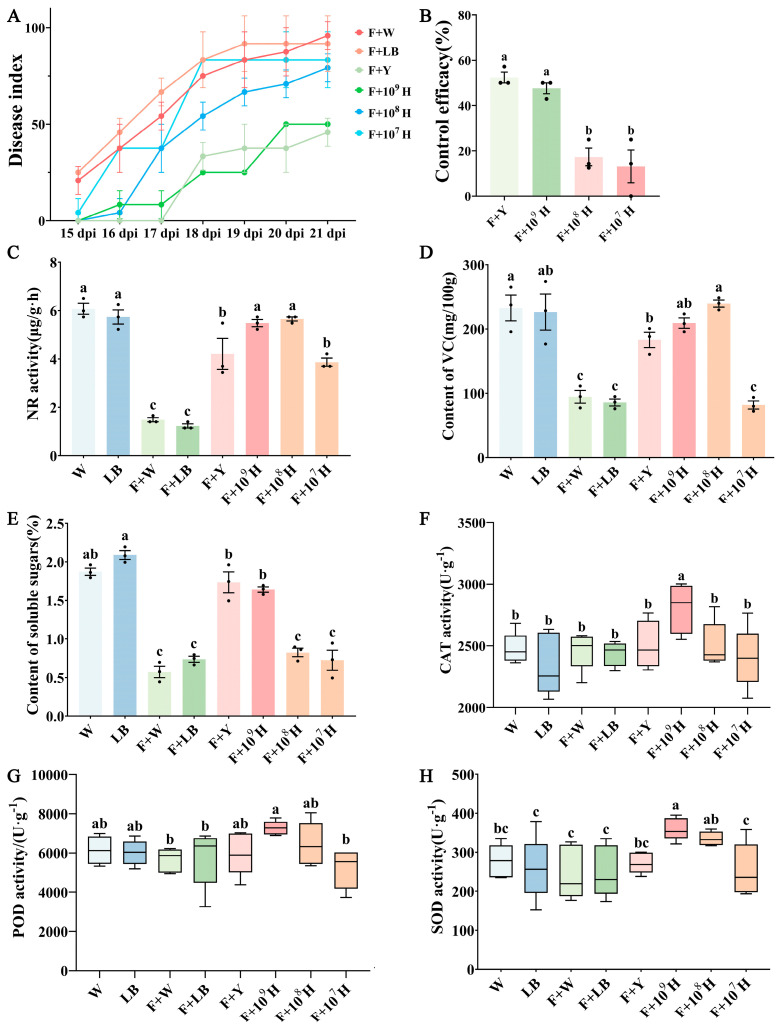
Biocontrol efficacy of HAB-2 in pot experiments and changes in defense-related enzyme activities in cowpea leaves. (**A**) Disease severity index; (**B**) Control efficacy; (**C**) Nitrate reductase activity; (**D**) VC content; (**E**) Soluble sugar content; (**F**) Catalase (CAT) activity; (**G**) Peroxidase (POD) activity; (**H**) Superoxide dismutase (SOD) activity. Each treatment was performed in triplicate. Data are presented as mean ± standard error (SE). Different letters indicate statistically significant differences among treatments at *p* < 0.05 based on ANOVA. (**A**,**B**) Each treatment was repeated three times, and each replicate contained three cowpea seedlings. (**C**,**D**,**E**) Each treatment was conducted with three replicates. (**F**,**G**,**H**) Each treatment included five replicates.

At 21 days post-treatment, the disease index in the group treated with 1 × 10^9^ CFU/mL HAB-2 was significantly reduced compared to the control group (Figure 4A), with a control efficacy of 47.62%. This was statistically comparable to the chemical fungicide treatment group (*p* < 0.05; Figure 4B). At this concentration, treated plants did not differ significantly from healthy plants (*p* > 0.05; Figure 4C–E) in physiological indicators, whereas growth parameters were significantly greater than those of the F + W and F + LB groups (Table S1). At 1 × 10^8^ CFU/mL, HAB-2 delayed symptom progression but did not significantly reduce the final disease index, while 1 × 10^7^ CFU/mL treatment showed no disease suppression (Figure 4A). To evaluate the ability of HAB-2 to induce disease resistance in cowpea, the leaf activities of defense-related enzymes were determined for each treatment group. At a concentration of 1 × 10^9^ CFU/mL, HAB-2 significantly increased CAT, SOD, and POD activities compared with the *F. oxysporum* f. sp. *tracheiphilum* AIQBFO93 treatment group (*p* < 0.05; Figure 4F–H). In contrast, treatments with HAB-2 at 1 × 10^8^ CFU/mL or 1 × 10^7^ CFU/mL showed no marked increases in CAT or POD activities (Figure 4F,G). Moreover, CAT, POD, and SOD activities in the hymexazol treatment group did not differ significantly from those of the control group (Figure 4F–H). The results indicate that the 1 × 10^9^ CFU/mL HAB-2 suspension provides effective biocontrol against CFW.

### 3.3. Isolation and Identification of Bacteria for Combination with B. velezensis HAB-2

Five bacterial strains with potential plant growth-promoting properties were isolated from the rhizosphere of cowpea. To determine their taxonomic status, phylogenetic analysis was performed based on their 16S rRNA gene sequences. Multiple sequence alignment was conducted using the MUSCLE algorithm, and a phylogenetic tree was constructed using the maximum likelihood method implemented in MEGA version 7.2, applying the Hasegawa-Kishino-Yano (HKY) model with invariant sites (I). The robustness of the tree topology was evaluated through 1000 bootstrap replications. Based on this analysis, the five isolates were identified as *P. putida* HD31, *P. hunanensis* HD33, *Priestia megaterium* HD36, *Stenotrophomonas maltophilia* HD41, and *E. soli* HD42. (Figure S1). These strains exhibited various plant growth-promoting traits (Table 2; Figures S2 and S3). Nitrogen fixation was observed in all five strains. HD33 and HD42 were also capable of producing siderophores, while HD42 further demonstrated phosphate solubilization and IAA production. Notably, HD41 also produced IAA, but its yield (17.89 ± 0.51 µg/mL) was 42% of that of HD42 (38.18 ± 0.62 µg/mL). HD33 and HD42 showed no direct antagonistic activity against *F. oxysporum* f.sp. *tracheiphilum* AIQBFO93 in a dual-culture assay. Additionally, no clear zones were observed on protease, cellulase, or chitinase detection media, indicating a lack of these enzymatic activities (Figure S4). Compatibility assays showed that HAB-2 had no antagonistic effects against HD33 or HD42, indicating mutual compatibility between the strains. Based on their individual traits and compatibility, HD33 and HD42 were combined with HAB-2 for subsequent biocontrol efficacy studies.

### 3.4. Control Efficacy of B. velezensis HAB-2 Combined with HD33 and HD42 Against Cowpea Fusarium Wilt

**Figure 5:**
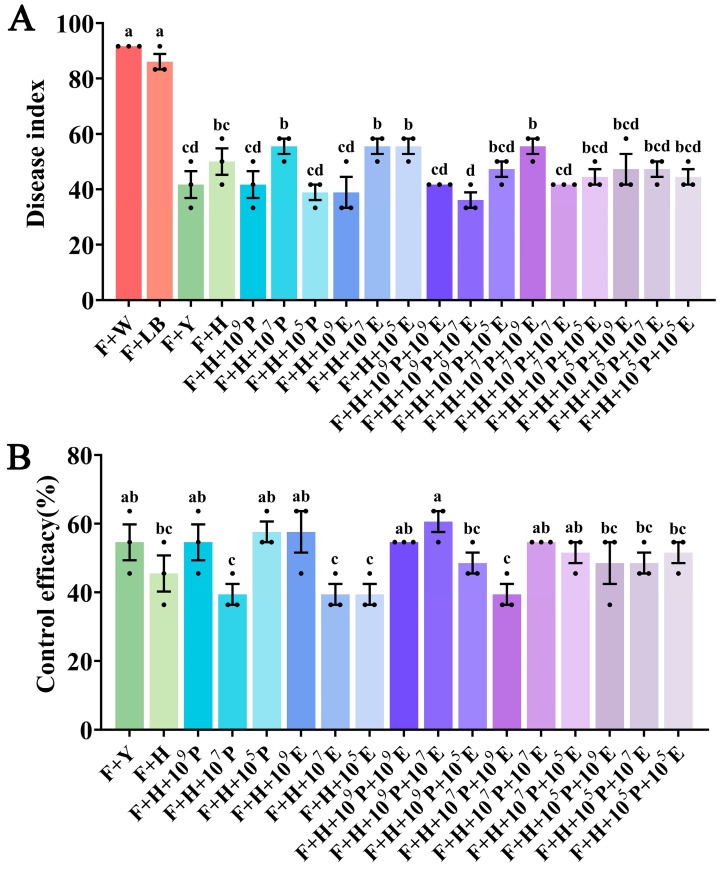
Biocontrol effects of the combined application of bacterial strains against cowpea Fusarium wilt. (**A**) Disease severity index; (**B**) Biocontrol efficacy. Each treatment was performed in triplicate, and data are expressed as mean ± standard error (SE). Different letters indicate statistically significant differences among treatments at *p* < 0.05 based on ANOVA. Each treatment was repeated three times, and each replicate contained three cowpea seedlings.

After 21 days, all combination treatments significantly reduced disease severity compared to the pathogen-only control (Figure 5A). Notably, the F + H + 10^5^P, F + H + 10^9^E, and F + H + 10^9^P + 10^7^E groups exhibited 12.12%, 12.12%, and 15.15% greater disease suppression, respectively, than HAB-2 alone (Figure 5B and Figure S5). These treatments also resulted in increased fresh weight, leaf area, plant height, and root length compared to other groups (Table S2), indicating that HD33 and HD42 can enhance the biocontrol efficacy of HAB-2 when applied at optimal ratios. Among these, the F + H + 10^9^P + 10^7^E treatment showed the most pronounced growth promotion, with fresh weight, leaf area, and plant height increased by 10.60%, 8.04%, and 7.81%, respectively, compared to the control group. In contrast, when HAB-2 was combined with HD33 or HD42 individually at a concentration of 1 × 10^7^ CFU/mL or lower, the biocontrol efficacy was inferior to that of HAB-2 alone, suggesting a concentration-dependent synergy. Co-application of HAB-2 with both HD33 and HD42 yielded more consistent results, although the F + H + 10^7^P + 10^9^ group showed a 6.06% reduction in efficacy. These findings highlight the importance of combining HAB-2 with HD33 and HD42 for effective biocontrol of CFW.

### 3.5. Analysis of Defense-Related Gene Expression in Cowpea Leaves

**Figure 6:**
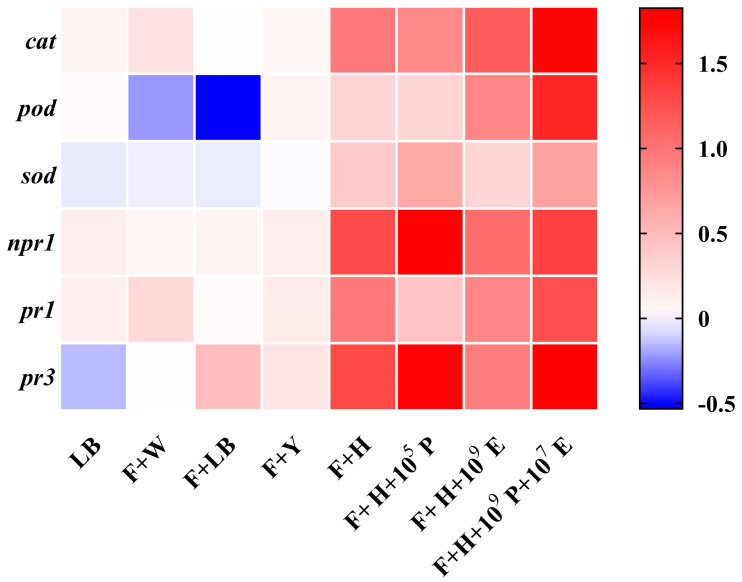
Expression profiling of defense-related genes in cowpea under different treatments.

Quantitative real-time PCR (RT-qPCR) was employed to assess the expression levels of defense-related genes in cowpea leaves treated with the three most effective combination treatments (F + H + 10^5^P, F + H + 10^9^E, and F + H + 10^9^P + 10^7^E), as well as with HAB-2 alone. Compared to the untreated control, HAB-2 treatment and combination treatments significantly upregulated the expression of *cat*, *pod*, and *sod*, consistent with the observed increases in CAT, POD, and SOD enzyme activities in the pot experiment. Additionally, the expression levels of *npr1*, *pr1*, and *pr3* were elevated in all the treatment groups (Figure 6). Compared to the HAB-2 alone treatment, the combination treatments enhanced the induction of different defense genes to varying degrees. Notably, the specific combination F + H + 10^9^ + 10^7^ demonstrated a stronger priming effect on defense markers than either HAB-2 alone or other combinations. These results demonstrate that the combinations of multiple strains represent a more effective strategy for activating plant defense responses compared to a single-strain application.

## 4. Discussion

Cowpea Fusarium Wilt is a globally prevalent soil-borne disease that impacts cowpea production, creating substantial obstacles to the growth of the cowpea industry [6]. As an environmentally friendly and sustainable approach, biological control has emerged as a viable strategy for managing Fusarium wilt [49]. *B. velezensis* is acknowledged as an important biocontrol agent due to its broad-spectrum antagonistic activity against a range of plant pathogens. This species serves as a critical resource in the development of microbial biofungicides and has found extensive applications in agricultural practices [50]. However, its practical application may still be limited by environmental conditions, especially within complex soil ecosystems where the functional performance of a single strain can be unpredictable, resulting in fluctuating field efficacy [22]. In contrast, microbial combinations exhibit enhanced adaptability to diverse environmental conditions, owing to the complementary functions and cooperative interactions among their component strains [31,50,51]. This study focuses on evaluating the application potential of a microbial consortium composed of *B. velezensis* HAB-2 and PGPR in controlling CFW. Understanding the biocontrol efficacy and underlying mechanisms of this consortium is of great significance for developing sustainable plant disease management strategies.

*B. velezensis* is known to produce a variety of antagonistic compounds such as enzymes, bacteriocins, lipopeptides, and volatile substances [52]. Jin et al. [53] have demonstrated that strain HAB-2 is capable of synthesizing cyclic lipopeptides, which have inhibitory effects on various pathogens. Further comparative genomic analysis has revealed the potential of this strain to produce numerous antimicrobial secondary [54]. This study found that HAB-2 secretes protease and cellulase, suppressing pathogens by disrupting their cell wall integrity.

Effective colonization of the plant rhizosphere is a critical prerequisite for PGPR to implement growth-enhancing and biocontrol functions [55]. Root colonization of cowpea by HAB-2 was confirmed through experimental observation. The observed colonization could be attributed to the strain’s cellulase production capability, given that certain microbes actively infiltrate plant tissues through cell wall-degrading enzymes such as cellulase and pectinase [56,57,58]. Successful colonization by rhizobacteria triggers interaction between their metabolites and plant immune pathways, subsequently activating transcription factors like *WRKY33* and inducing broad-spectrum systemic resistance against pathogens [59]. In the present study, colonization of cowpea roots by HAB-2 led to increased activities of defense-related enzymes (CAT, SOD, POD) in leaves, and a corresponding upregulation in the expression of their associated genes (*cat*, *sod*, *pod*). Concurrently, the pathogenesis-related protein genes (*pr1*, *pr3*) and their upstream regulatory gene *npr1* were also upregulated, collectively enhancing plant defense responses. This suggests that HAB-2 induces systemic resistance via a mechanism akin to other *Bacillus* species, wherein root colonization activates defense not only at the infection site but also in distant tissues, thereby offering protection against both root and foliar pathogens.

Effective colonization of plant roots by *Bacillus* strains is a key determinant of their biocontrol performance. In this study, a high concentration of *B. velezensis* HAB-2 (1 × 10^9^ CFU/mL) significantly suppressed the development of CFW, with a control efficacy of 47.62%. Although hymexazol showed slightly higher efficacy (52.38%), the difference was not statistically significant. It is noteworthy that HAB-2 demonstrated a clear dose-dependent effect, lower concentrations (1 × 10^7^ CFU/mL) did not effectively suppress the disease. This limitation may be attributed to insufficient microbial density, which was inadequate to overcome niche competition, thereby restricting successful root colonization under natural conditions [60,61]. This underscores the importance of developing high-concentration biocontrol formulations for practical application.

Previous research has substantiated that multi-strain formulations can overcome the limitations of individual strains, thereby enhancing the efficacy of plant disease control [31,62,63,64]. A comparative field study revealed that the compound microbial agent combined with carbendazim (CM) provided the most effective control of banana *Fusarium* wilt, with an efficacy of 60.53%. Furthermore, this treatment significantly modified the composition and function of the rhizosphere bacterial community by enriching beneficial genera such as *Bacillus* and *Pseudomonas* during both the vegetative growth and fruiting stages [65]. Furthermore, the optimized co-cultivation of three *Bacillus* strains—*Paenibacillus polymyxa* PJH16, *B. velezensis* VJH504, and *B. subtilis* JNF2 were further validated through pot experiments, which confirmed their synergistic efficacy against cucumber *Fusarium* wilt, achieving a control rate of 93.4%. This significantly surpassed the performance of single-strain treatments and chemical fungicides [63]. Two PGPR strains, *P. hunanensis* HD33 and *E. soli* HD42, were isolated from the rhizosphere soil of cowpea treated with HAB-2 and found to be biologically compatible with it. Both strains have been previously reported to possess plant growth-promoting properties [66,67]. In this study, *P. hunanensis* HD33 demonstrated nitrogen-fixing and siderophore-producing capabilities, akin to the growth-promoting traits of HAB-2. *E. soli* HD42 exhibited more diverse rhizosphere-promoting traits, including solubilizing inorganic phosphate and synthesizing IAA. Collectively, the combination of these three strains was expected to possess a range of functional traits, including nitrogen fixation, phosphate solubilization, IAA production, and siderophore biosynthesis, in addition to antagonistic potential against the CFW pathogen. QPCR analysis revealed that specific bacterial combinations upregulated defense-related genes more strongly than individual strains, indicating an enhanced induced systemic resistance. Pot experiments substantiated the synergistic biocontrol and growth-promoting effects of this combination. When compared to HAB-2 alone, the combined treatment improved disease control by 15.15%, and increased fresh weight, leaf area, and plant height by 10.60%, 8.04%, and 7.81%, respectively, underlining the synergistic enhancement among strains. Notably, dual-strain combinations exhibited lower efficacy than HAB-2 alone at certain concentrations, whereas the triple-strain combination demonstrated greater stability and performance. This indicates that multi-strain combinations may enhance biocontrol performance through diverse and complementary mechanisms. Taken together, these results indicate that this combination exhibits robust biocontrol efficacy and plant growth-promoting traits under various conditions. Its consistent performance across different concentrations underscores its potential as a promising candidate for sustainable cowpea cultivation. Further investigations are warranted to clarify its underlying molecular mechanisms and field-level applicability.

## 5. Conclusions

HAB-2 is capable of producing protease and cellulase, which inhibit the mycelial growth and sporulation of the *F. oxysporum* f. sp. *tracheiphilum* AIQBFO93. It exhibits efficient root colonization and activates systemic resistance mechanisms in host plants. Pot experiments have demonstrated the biocontrol activity of HAB-2 in managing CFW, with a control rate of up to 47.62%. The synergistic application of HAB-2 with *P. hunanensis* HD33 and *E. soli* HD42 provided superior and more consistent control of CFW compared to single-strain treatments, with a 15.15% improvement over HAB-2 alone. Moreover, the combined treatment significantly increased fresh weight (10.60%), leaf area (8.04%), and plant height (7.81%), and further enhanced the expression of defense-related genes in cowpea. The combinations of these three strains provided superior and more consistent control of CFW compared to single-strain applications, as demonstrated by pot experiments. The enhanced efficacy of the combination is likely a result of combined effects: (1) the direct plant growth-promoting (PGP) traits of strains HD33 and HD42, which may improve overall plant health, and (2) the synergistic effect between the strains that more effectively primes the plant’s defense system than HAB-2 alone. However, the underlying mechanisms require further investigation to be fully elucidated. HAB-2, in combination with *P. hunanensis* HD33 and *E. soli* HD42, exhibited notable synergistic biocontrol and growth-promoting potential in pot experiments. This study validated the effectiveness of the microbial consortium through in vivo assays, providing a solid theoretical and experimental foundation for its application in agronomic practice. Although further field trials are required to assess its stability and adaptability under diverse conditions, the current findings strongly suggest that this microbial combination holds practical promise as a sustainable strategy for managing cowpea *Fusarium* wilt and merits further exploration and promotion in agricultural production.

## Figures and Tables

**Table 1:** Primers used in the RT-qPCR experiment.

Gene Name	Accession	Forward Primer	Reverse Primer
*cat*	XM_028047190.1	GTCTCAGGCTGACCGTTCTC	CTAACACACAAACGGCATCG
*pod*	XM_028078850.1	TGTCTGGAGGTCCTTCGTG	AGGTTGAGTTCAGGGTTGG
*sod*	XM_028076317.1	CAATGGTTGCCTGTCAACTG	AACAGCTCTCCCAATGATGG
*npr1*	XM_028075607.1	TTGGACTCCGATGATGTTGA	GCAGCAACATGAAGCACTGT
*pr1*	QCD76639.1	TGCTTTCGCACAAAACTACG	GTCTGCACTCTCCACCAACA
*pr3*	BAA77691.1	TGAGGCAAACTTGGGATACAG	CTTCGTTGGGTGGAGCA
*EF1-α*	XM_007151727.1	GGTCATTGGTCATGTCGACTCTG	GCACCCAGGCATACTTGAATGAC

**Table 2:** Detection of plant growth-promoting traits among isolated bacterial strains.

Strains	Nitrogen Fixation	Phosphate Solubilization (Inorganic, Organic Forms)	Solubilization of Potassium	Siderophores	IAA
HD31	+	−,−	−	−	−
HD33	+	−,−	−	+	−
HD36	+	−,−	−	−	−
HD41	+	−,−	−	−	+
HD42	+	+,−	−	+	+

“+” indicates that the strain tested positive for the corresponding trait; “−” indicates a negative result.

## Data Availability

The original contributions presented in this study are included in the article/Supplementary Material. Further inquiries can be directed to the corresponding author.

## Supplementary Materials

The following supporting information can be downloaded at: https://www.mdpi.com/article/10.3390/microorganisms13112578/s1, Figure S1: Maximum likelihood phylogenetic tree of five soil-derived bacterial isolates based on 16S rRNA sequences; Figure S2: Plant growth-promoting trait assays of five soil-derived bacterial isolates; Figure S3: Indole-3-acetic acid (IAA) production by five soil-derived bacterial isolates; Figure S4: Determination of extracellular enzyme activities and antifungal effects of HD33 and HD42 against *F. oxysporum* f. sp. *tracheiphilum* AIQBFO93. Figure S5: Representative images of cowpea plants in pot experiments. Representative images of cowpea plants in pot experiments. Table S1: Growth performance of cowpea under different concentrations of HAB-2 treatment; Table S2: Growth performance of cowpea following treatment with biocontrol strain combinations.
