# Supplementary material for: Evaluation of Biocontrol Efficacy of Bacillus velezensis HAB-2 Combined with Pseudomonas hunanensis and Enterobacter soli Against Cowpea Fusarium Wilt
Source: Microorganisms. 2025 Nov 12;13(11):2578. doi: 10.3390/microorganisms13112578 (PMC12654332; doi:10.3390/microorganisms13112578)
Supplement: Supplementary file 1 [file microorganisms-13-02578-s001.zip › microorganisms-3897704-supplementary.pdf]

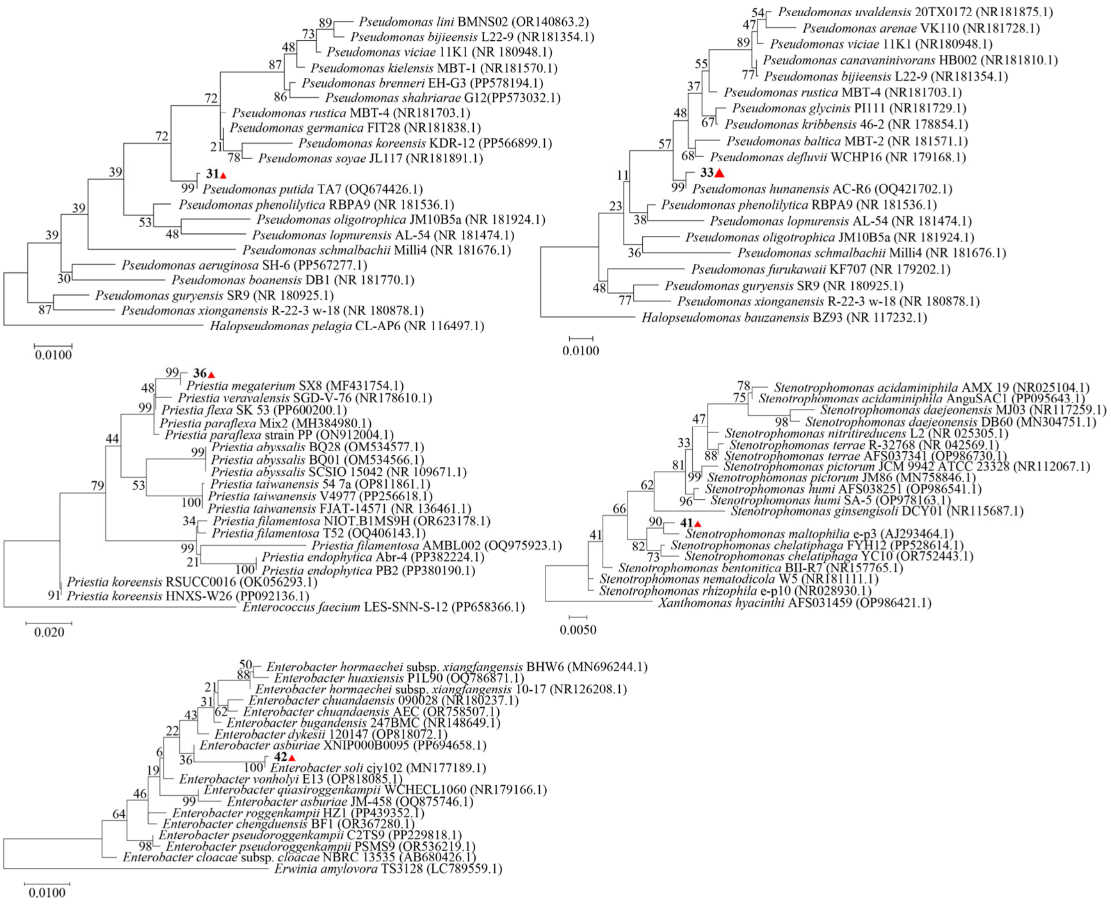

Figure S1. Maximum likelihood phylogenetic tree of five soil-derived bacterial isolates based on 16S rRNA sequences.

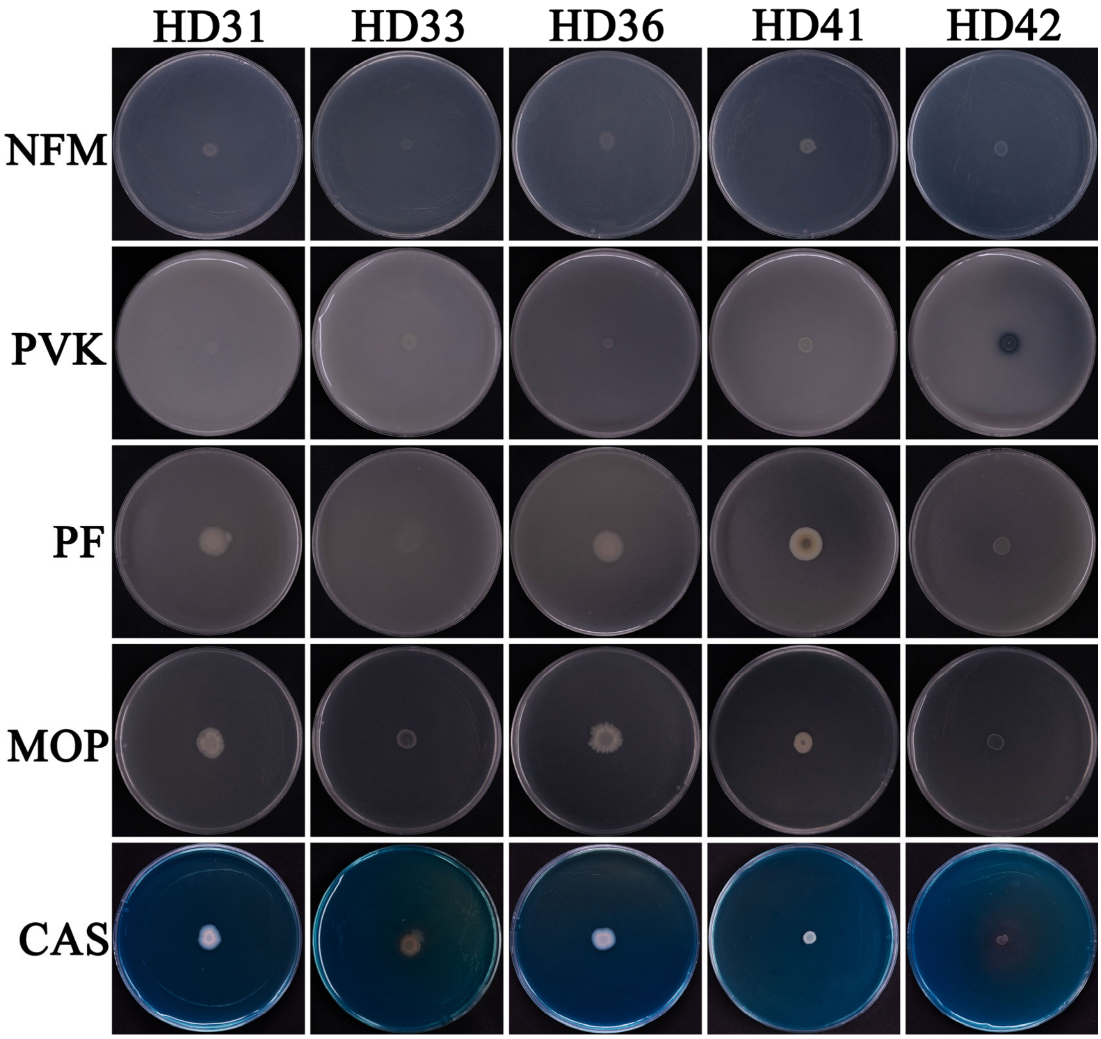

Figure S2. Plant growth-promoting trait assays of five soil-derived bacterial isolates.

NFM, Nitrogen-free agar medium. PVK, MeHKNHa inorganic phosphorus medium. PF, potassium feldspar agar medium. MOP, MeHKNHa organic phosphorus medium. CAS, Chrome Azurol S agar medium.

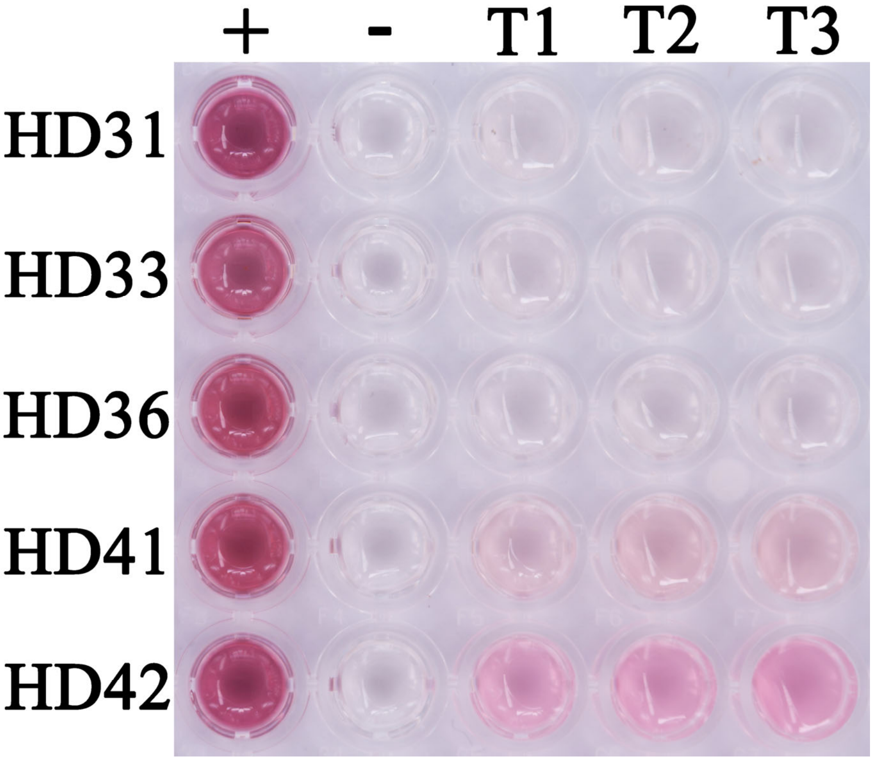

Figure S3. Indole-3-acetic acid (IAA) production by five soil-derived bacterial isolates.

"+" indicates the positive control, "−" indicates the negative control, and T1–T3 represent the treatment groups.

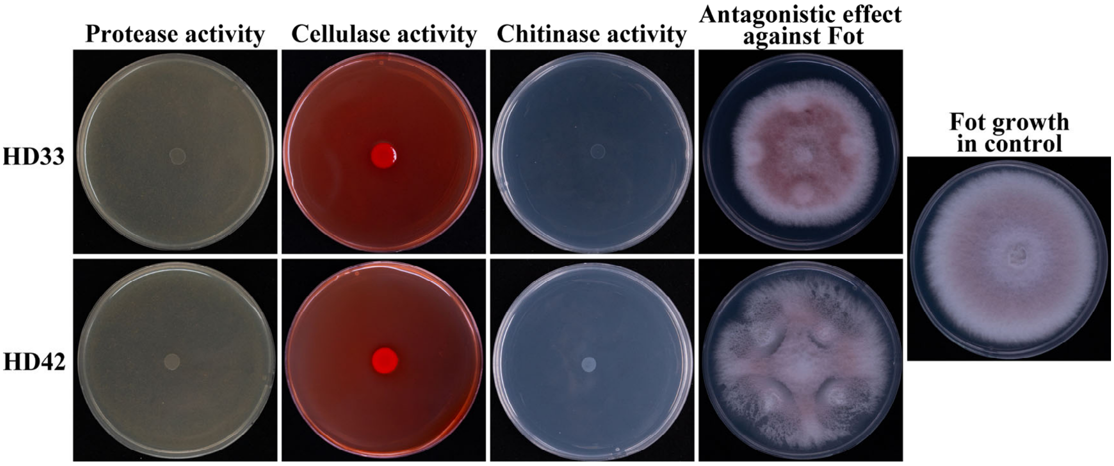

Figure S4. Determination of extracellular enzyme activities and antifungal effects of HD33 and HD42 against Fot

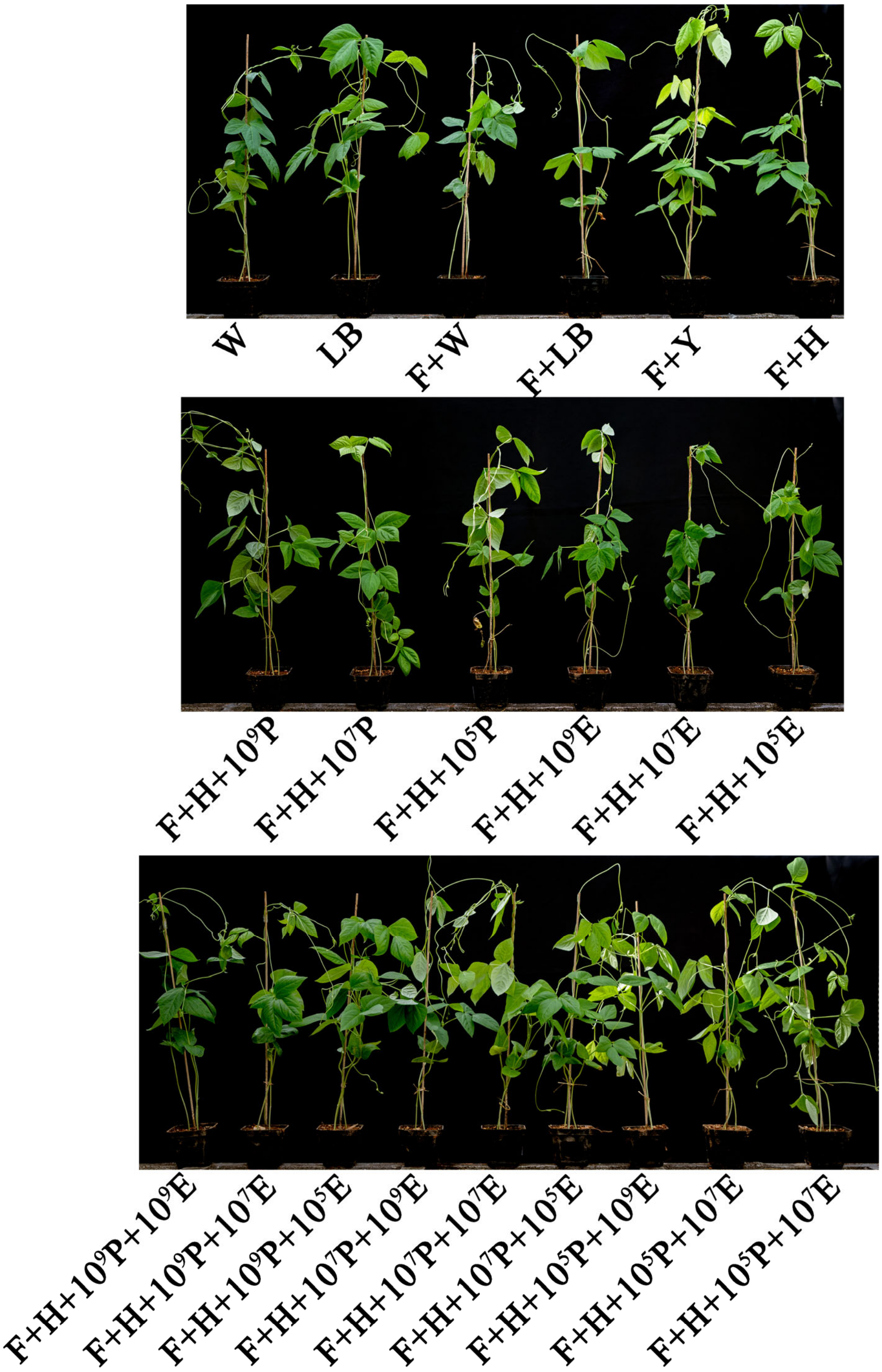

Figure S5. Representative images of cowpea plants in pot experiments.

Table S1. Growth performance of cowpea under different concentrations of HAB-2 treatment.

Treatment	Fresh weight/g	leaf surface area/mm^2	Root length/cm	Plant height/cm
W	1.56±0.37^{ab}	4112.51±220.34^{a}	4.69±0.54^{ab}	22.03±0.76^{a}
LB	1.74±0.68^{a}	4005.60±306.51^{ab}	4.71±0.52^{a}	19.57±1.34^{ab}
F+W	1.01±0.11^{bc}	2444.93±160.57^{c}	3.02±0.51^{c}	15.87±0.70^{cde}
F+LB	1.00±0.20^{bc}	2488.89±187.10^{c}	3.37±0.35^{abc}	14.31±0.78^{e}
F+Y	1.28±0.29^{abc}	3709.9±75.96^{a}	3.46±0.31^{abc}	18.02±0.86^{bcd}
F+10^{9}H	1.41±0.15^{abc}	3465.86±154.85^{b}	4.65±0.45^{bc}	19.13±0.71^{abc}
F+10^{8}H	1.27±0.14^{abc}	2657.59±147.98^{abc}	2.78±0.34^{c}	18.45±0.71^{bcd}
F+10^{7}H	0.97±0.06^{c}	2439.30±37.80^{c}	3.19±0.68^{abc}	15.68±2.20^{de}

Different letters indicate statistically significant differences among treatments at $p < 0.05$ based on ANOVA.

Table S2. Growth performance of cowpea following treatment with biocontrol strain combinations.

Treatment	Fresh weight/g	Leaf surface area/mm^2	Root length/cm	Plant height/cm
W	8.11±0.86^{abc}	18834.87±1000.21^{abc}	13.50±3.52^{a}	109.05±6.94^{abc}
LB	8.68±1.74^{ab}	19599.33±1851.94^{ab}	12.49±5.61^{ab}	109.47±7.99^{abc}
F+W	5.91±0.22^{d}	11784.61±520.88^{g}	6.41±0.62^{d}	89.87±5.71^{d}
F+LB	5.81±0.59^{d}	11487.09±895.54^{g}	6.42±0.65^{d}	89.52±7.42^{d}
F+Y	7.35±0.73^{abcd}	17366.67±391.56^{cde}	11.30±2.16^{abc}	110.08±3.41^{abc}
F+H	7.56±0.71^{abcd}	17295.24±151.88^{cde}	11.86±1.59^{abc}	112.20±5.40^{abc}
F+H+10^9P	7.51±0.79^{abcd}	18296.57±313.61^{abc}	9.54±2.61^{bcd}	113.70±3.41^{ab}
F+H+10^7P	7.70±0.77^{abcd}	15813.44±294.45^{def}	8.34±0.90^{cd}	106.95±3.72^{abc}
F+H+10^5P	8.16±0.25^{abc}	20220.10±454.62^{a}	9.54±1.20^{bcd}	115.76±2.40^{a}
F+H+10^9E	8.32±0.91^{abc}	19223.35±632.19^{abc}	9.74±0.68^{bcd}	113.84±9.52^{ab}
F+H+10^7E	6.28±0.44^{cd}	14807.89±992.60^{f}	8.42±0.50^{cd}	96.61±3.18^{cd}
F+H+10^5E	6.53±0.63^{cd}	14365.35±245.22^{f}	8.62±0.35^{cd}	97.19±3.58^{cd}
F+H+10^9P+10^9E	8.37±2.46^{abc}	18577.26±538.29^{abc}	11.36±1.08^{abc}	112.13±9.01^{abc}
F+H+10^9P+10^7E	8.92±0.60^{a}	20350.01±442.77^{a}	10.47±0.18^{abc}	117.57±11.97^{a}
F+H+10^9P+10^5E	8.97±1.88^{a}	15241.78±1122.19^{ef}	10.50±0.78^{abc}	116.40±3.61^{a}
F+H+10^7P+10^9E	7.43±1.47^{abcd}	15625.38±331.38^{ef}	10.45±0.74^{abc}	97.09±0.34^{cd}
F+H+10^7P+10^7E	6.69±0.35^{bcd}	15677.93±524.55^{ef}	9.24±0.35^{bcd}	99.25±3.84^{bcd}
F+H+10^7P+10^5E	6.64±1.54^{bcd}	17853.16±478.92^{bcd}	9.69±1.53^{bcd}	102.54±1.16^{abcd}
F+H+10^5P+10^9E	6.64±0.26^{bcd}	18535.40±459.33^{abc}	9.70±1.03^{bcd}	107.93±13.05^{abc}
F+H+10^5P+10^7E	6.46±0.51^{cd}	17944.33±673.16b^{cd}	9.33±0.71^{bcd}	109.55±4.48^{abc}
F+H+10^5P+10^5E	6.77±0.41^{bcd}	16051.82±526.88^{def}	8.72±0.36^{cd}	104.82±10.77^{abcd}

Different letters indicate statistically significant differences among treatments at $p < 0.05$ based on ANOVA.
